# In vitro affinity maturation of antibody against membrane-bound GPCR molecules

**DOI:** 10.1007/s00253-019-10030-x

**Published:** 2019-07-29

**Authors:** Jie Wang, Lili An, Yun Zhao, Cheng Zhang, Shengnan Li, Chen Ye, Shuqian Jing, Haiying Hang

**Affiliations:** 1 Key Laboratory for Protein and Peptide Pharmaceuticals, National Laboratory of Biomacromolecules, Institute of Biophysics, Chinese Academy of Sciences, Beijing, 100101 China; 2 University of Chinese Academy of Sciences, Beijing, 100039 China; 3 Gmax Biopharm LLC, Hangzhou, 310052 China

**Keywords:** GPCR, CHO cell display, Vesicle, Affinity maturation, ETaR

## Abstract

G protein-coupled receptors (GPCRs), also known as seven-transmembrane domain receptors, are among the most important targets against which many small molecule drugs have been developed. However, only two antibody drugs targeting GPCRs have been approved for clinical use although many antibody drugs against non-GPCR protein targets have been successfully developed for various disease indications. One of the challenges for developing anti-GPCR drugs is the high difficulty to perform affinity maturation due to their insolubility in aqueous solutions. To address this issue, CHO cell display libraries of single-chain variable fragments (scFvs) and full-length antibodies were maturated directly against vesicle probes prepared from CHO cells displaying the endothelin A receptor (ETaR) GPCR. The probe in the vesicle form ensures the physiological conformation and functional activity of the protein and avoids issues with membrane protein insolubility. The size of the vesicle had a clear effect on protein-ligand interaction; we used small-sized vesicles with low expression levels of GPCRs for the affinity maturation. Four rounds of affinity maturation combining vesicles as probes with the CHO cell display platform improved affinity by 13.58-fold for scFvs and 5.05-fold for full-length antibodies. We expect that this method will not only be used for the affinity maturation of antibodies against GPCRs but will also be used to mature antibodies for other types of proteins where the conformation/activity of which depends on the proper membrane environment.

**Electronic supplementary material:**

The online version of this article (10.1007/s00253-019-10030-x) contains supplementary material, which is available to authorized users.

## Introduction

In recent decades, antibodies have become more and more important in therapeutics, as evidenced by an increasing number of FDA-approved monoclonal antibodies (Schrama et al. 2006). The advantages of antibody drugs over small-molecule drugs include superior specificity, prolonged serum half-life, and high druggability (Hutchings et al. 2010). It is generally considered that only the affinity of monoclonal antibodies up to 1 nM or higher to their target antigens will reach the requirement of clinical development (Carlin et al. 1999; Maynard et al. 2002; Putnam et al. 2008; Wu et al. 2007). In vitro affinity is needed when the affinity of antibodies generated by immunizing animals or screening antibody libraries does not meet the requirement for drug development. Moreover, to reduce their antigenicity, humanization of antibodies generated from non-humanized animals are needed, which frequently results in reduction of antibody affinity (Makabe et al. 2008; Verhoeyen et al. 1988). Therefore, affinity maturation of antibodies in vitro is necessary for the generation of clinically usable antibody drugs (Schlapschy et al. 2008; Schlapschy et al. 2005).

GPCRs are seven-transmembrane domain receptors that play important roles in physiology and pathology (Alexander et al. 2013). GPCRs are the largest group of eukaryotic cell surface receptors, which mediate signal transduction initiating downstream cell signaling events triggered by a variety of stimulants, including light, odorant molecules, neurotransmitters, hormones, and growth factors and linked to a wide range of diseases such as cancer, inflammation, and metabolic diseases (Conn et al. 2007). Accordingly, GPCRs served as one class of the most important therapeutic targets, and approximately 34% of all currently marketed drugs are targeted to GPCRs (Hauser et al. 2017; Hauser et al. 2018; Hutchings et al. 2017; Raskandersen et al. 2011; Raskandersen et al. 2014; Santos et al. 2017). In spite of their importance in pathogeny and treatment, only two antibody drugs targeting GPCRs have been approved for clinical use. Aimovig is the first and only anti-GPCR antibody that received marketing approval of the FDA. It was engineered by Amgen for treatment of the migraine-targeting CGRP (calcitonin gene-related peptide) (Goldberg and Silberstein 2015). Before Aimovig, Mogamulizumab, also called Potelligent, was approved in Japan for the treatment of relapsed or refractory adult T cell leukemia-lymphoma (ATL) (Subramaniam et al. 2012). The development of GPCR-targeting antibody drugs has been beset by difficulties in the preparation of native and functional form of antigens as well as the lack of a suitable antibody-maturation platform for selectivity (Jo and Jung 2016).

Technologies for screening for or selecting antibodies in vitro include phage (De Bruin et al. 1999; Huse et al. 1992; Smith 1985; Winter et al. 1994), yeast (Boder and Wittrup 1997; Feldhaus et al. 2003), and bacterial (Francisco and Georgiou 2006; Mazor et al. 2009; Qiu et al. 2010) and mammalian cell displays (Chen et al. 2016). Although these techniques have been successful in obtaining tighter binders from libraries against free individual protein antigens, they are difficult to use for maturing antibodies targeting GPCRs or other membrane-bound proteins (Cho and Shusta 2010; Lipes et al. 2008). In recent years, there have been only limited improvements in the technologies for maturing GPCR antibodies. For instance, yeast display has been used to mature an anti-transferrin receptor (TfR) scFv by using detergent-solubilized cell lysates as probes (Tillotson et al. 2015). The problem is that detergent-solubilized protein probes may not guarantee to possess native conformations (Hansen et al. 2018; Hotzel et al. 2011; Wilkinson et al. 2015). Phage display has been used in affinity maturation for antibodies against GPCR molecules (CCR4, CC chemokine receptor 4 and formyl-peptide receptor 1) on plasma membrane of intact human cells (Hagemann et al. 2014; Krebs et al. 2001); thus, these GPCR molecules are in native conformations. However, in this procedure, each human cell binds to multiple phages with different antibodies and quantitative sorting cannot be applied, so it is labor-intensive. Therefore, new technologies that can efficiently mature antibodies against GPCR or other membrane-bound antigens are needed.

In humans, endothelin represents the most potent and long-lasting vasoconstrictor (Hillier et al. 2001). Endothelin, via activation of ETaR (a GPCR), contributes to the development of vascular disease such as hypertension and atherosclerosis (Barton 2000). Small-molecule drugs targeting ETaR has been used for patients with a range of vascular and nonvascular diseases (Casserly and Klinger 2008; Maneenil et al. 2017; Okamoto et al. 2016). However, these small-molecule drugs have serious side effects in patients (Hartman et al. 2010). One possible solution for the side effects of the small-molecule drugs is to develop an antibody blocker to ETaR. An antibody (80H4) developed by Gmax Biopharm LLC (Zhangzhou, China) is a specific binder and inhibits vasoconstriction. In this study, we used this antibody as a model to develop an affinity maturation protocol for an anti-GPCR antibody.

In this study, we propose a new technology platform to improve affinity of antibodies targeting GPCRs. We used small-size vesicles displaying GPCR molecules as probes and CHO cells to display antibodies. The vesicle format conserves native conformation of GPCR molecules. The size difference between vesicles and CHO cells provides an opportunity for each cell to bind multiple vesicles so as to distinguish antibody binding abilities. We have carried out the maturation of both scFv and full-length antibody against a GPCR (ETaR), and obtained antibody mutants with significantly higher affinities to ETaR.

## Materials and methods

### Construction of plasmids

The sequences of the primers for construction of the plasmids are listed in Table S1, and all the constructed plasmids below were confirmed by sequencing.

The dual recombinase expression plasmid pCI-Flp-2A-Cre (pF2AC) and the exchange plasmid pFRT-Ab-LoxP (pFAbL) were constructed previously in our lab (Chen et al. 2016). The plasmid pFAbL possessed a transmembrane domain (TM) so the antibody could be displayed on the cell surface (Chen et al. 2012). The anti-GPCR monoclonal antibody against ETaR (80H4) was originally generated by Gmax Biopharm LLC (Zhangzhou, China). The sequences of light-chain and heavy-chain variable regions were codon-optimized to maximize the transcription level using an in-house computer program from Genscript (Nanjing, China), and the codon-optimized DNAs were synthesized in the company. The DNA sequences of the anti-GPCR and its codon-optimized antibodies have been submitted to the EMBL Nucleotide Sequence Database with accession numbers LR590084-LR590085 and LR590086-LR590087, respectively.

The plasmid pFRT-anti-GPCR(80H4)-scFv for inserting the antibody into the host CHO cells was created in the following steps. First, anti-GPCR-HV (heavy chain region) was amplified by PCR using primers Overlap-scFv-P2 and Overlap-scFv-P4, and anti-GPCR-LV (light chain region)-HA was amplified by PCR using primers Overlap-scFv-P3 and scFv-*Xho*I-P5. Second, SP-anti-GPCR-HV-(G4S)_3_-LV-HA was amplified by overlap PCR using primers scFv-*Eco*R1-P1 and scFv-*Xho*I-P5, with a (G4S)_3_ linker connecting anti-GPCR-VH and anti-GPCR-VL together. Third, SP-anti-GPCR-scFv-HA cassette was inserted into the pFAbL plasmid between *Eco*RI and *Xho*I. The plasmid pFRT-anti-GPCR full-length was constructed in the following steps. Kozak (Kozak sequence)-SP (signal peptide)-anti-GPCR-LC (light chain constant region) was cloned into pFRT-Ab-dual-CMV-LoxP plasmid between the *Eco*RI and *Eco*RV sites to generate the plasmid pFRT-anti-GPCR-LC-dual-CMV-Loxp, with a double CMV promoter for the concurrent expression of both HC and LC of antibodies. Kozak-SP-anti-GPCR-HC (heavy-chain-constant region) was cloned into pFRT-anti-GPCR-LC-dual-CMV-Loxp plasmid between the *Nhe*I and *Xho*I sites for the generation of the pFRT-anti-GPCR-full-length plasmid.

For the expression and purification of the proteins used in this study, we constructed the following plasmids. pCEP4-anti-GPCR-scFv-His for scFv-His expression was constructed by inserting the SP-anti-GPCR-HV-(G4S)_3_-LV (described above) into the pCEP4 vector (Invitrogen, USA) between the *Hin*dIII and *Xho*I sites. We used pCDNA3.1(+) (Invitrogen, USA) to construct the two plasmids pCDNA3.1(+)-anti-GPCR-full-length-LC and pCDNA3.1(+)-anti-GPCR-full-length-HC for full-length antibody expression and purification. Kozak-SP-LV-LC was cloned into pCDNA3.1(+) between the *Eco*RI and *Xho*I sites, then Kozak-SP-HV-HC was cloned into pCDNA3.1(+) between the *Cla*I and *Xho*I sites to generate pCDNA3.1(+)-anti-GPCR-full-length-HC. We constructed pCEP4-anti-GPCR-scFv-Fc for purification of scFv-Fc format antibody by amplifying Kozak-SP-scFv-Fc by overlap PCR, then inserting it into pCEP4 between *Hin*dIII and *Xho*I.

We constructed the following two plasmids for the purification of the proteins in *E. coli*. The pET28a(+)-GFP plasmid was created by inserting a *EGFP* gene into the pET28a(+) plasmid between *Sac*I and *Xho*I. Similarly, we constructed the pET28a(+)-RFP plasmid by inserting a *RFP* gene into the pET28a(+) plasmid between *Bam*HI and *Hin*dIII.

We also constructed the plasmid pCEP4-PD1-Fc for the display of the PD1-Fc protein in CHO cells. The gene sequence of PD1 was obtained from the RCSB Protein Data Bank (http: //www.rcsb.org, PDB ID: 3RRQ). The plasmid pCEP4-PD1-Fc was created in the following steps. First, SP-PD1 was amplified by PCR using primers SP-*Hin*dIII-P1 and PD1-Overlap-P2, and Fc was amplified by PCR using primers Fc-Overlap-P3 and Fc-*Bam*HI-P4. Second, SP-PD1-Fc was amplified by overlap PCR using primers SP-*Hin*dIII-P1 and Fc-*Bam*HI-P4, with a (G4S)_3_ linker connecting SP-PD1 and Fc together. Third, SP-PD1-Fc was inserted into the pCEP4 plasmid between *Hin*dIII and *Bam*HI.

For preparation of vesicles expressing PD-L1-GFP, we constructed the plasmid PCEP4-PD-L1-GFP-TM by inserting the open frame of the *PD-L1* gene into PCEP4 between *Hin*dIII and *Xho*I. The plasmids ETaR-GFP and ETaR were gifts from Gmax Biopharm LLC (Hangzhou, China).

### Cell culture

CHO/dhFr^−^ cells (12200036, Cell Bank of the Chinese Academy of Sciences, Shanghai, China) and the cell lines derived from them were propagated in IMDM medium (HyClone) containing 10% fetal bovine serum (HyClone), 0.1 mM hypoxanthin, and 0.016 mM thymidine (HT, Gibco, USA), at 37 °C in a 5% CO_2_ incubator. The suspension Expi293F cells (Invitrogen, Carlsbad, CA, USA) were cultured in SMM 293-TII medium (Sino BiologicalInc, Beijing, China) in suspension at 37 °C, 5% CO_2_. Cell density was maintained between 3 × 10^5^ and 3 × 10^6^ cells/ml by dilution of the cell suspension in the same growth medium.

### Preparation of vesicles

A modified method reported by Hang et al. (Haiying et al. 1990) was used to obtain vesicles displaying ETaR. ETaR-GFP cells were detached from dishes using 2.5 mM EDTA-PBS, then collected by centrifuging at 300×*g* for 3 min and washed with 5 ml ice-cold 20 mM Hepes buffer (pH 7.3). Subsequently, cells were suspended in Hepes buffer at a density of about 5 × 10^7^ cells/ml for cell vesicle preparation; this and all subsequent steps were performed on ice or at 4 °C. Proteinase inhibitor (Roche, Germany, 04693159001) mixture was added to the cell suspension to avoid protein degradation. The cell homogenization and cell membrane preparation were performed by following the procedure reported by Hang et al. (Haiying et al. 1990). The harvested cell membrane vesicles were suspended in 1 ml opti-MEM and stored in a refrigerator at 4 °C. The average diameter of these vesicles was 200 nm. We used the Mini-Extruder Set (Avanti, 610000) to prepare vesicles smaller than 200 nm, gently pushing the above-described vesicles through a PC membrane with a designated pore size between the two syringes 11 times.

### Transfection and stable cell line establishment

To prepare the cells displaying PD1-Fc proteins and affinity-matured PD1-Fc proteins, CHO cells were seeded 24 h prior to transfection to achieve 80% confluence in a 6-well plate and transfected with 1 μg wild-type or affinity-matured PD1-Fc plasmids (pCEP4-PD1-Fc or pCEP4-matured PD1-Fc) using the Lipofectamine™ 2000 (Invitrogen) following the manufacturer’s recommendations. Forty-eight hours after transfection, the cells were detected by a flow cytometer.

To generate cells displaying scFv and full-length anti-GPCR (ETaR), these two antibody genes from the plasmids PFRT-anti-GPCR-scFv and PFRT-anti-GPCR-full-length were integrated into the PuroR genome site of PuroR-12 CHO cells (Chen et al. 2016) by following a procedure reported by Chen et al. (2016). The cells that displayed the highest levels (the top 1%) of the antibody were flow-sorted and harvested for later use.

The two CHO cells stably expressing and displaying ETaR and ETaR-GFP were provided by Gmax Biopharm LLC (Zhangzhou, China).

### PCR amplification

PCR for cloning genes was carried out using pyrobest DNA polymerase (Takara) (94 °C for 3 min; 30× 94 °C for 30 s, 58 °C for 30 s, 72 °C for 3 min; 72 °C for 10 min), while PCR for antibody gene sequencing was carried out using a high-fidelity PCR kit (NEB) (98 °C for 3 min; 30× 98 °C for 30 s, 58 °C for 30 s, 72 °C for 3 min; 72 °C for 10 min). The cloned genes were confirmed by sequencing.

### Antibody affinity maturation

To mature antibody affinity, CHO cells that displayed single-chain or full-length anti-GPCR antibodies were seeded into a 6-well plate. The cells were transfected with 2 μg of pCEP4-Neo-AID (activation-induced cytidine deaminase) (Chen et al. 2016) and 5 μl of Lipofectamine 2000 for 5 h, washed and maintained in IMDM containing 10% FBS and HT for 1 day, then the cells were expanded in IMDM with 10% FBS, HT, 1 mg/ml G418 for 7 days and flow-sorted for cells that expressed high affinity antibodies.

### Antibody gene sequencing

The genomic DNA of the cells was extracted with a genomic DNA purification system (Promega), and the scFv genes were PCR amplified using primers scFv-CMV-forward: 5-CGCAAATGGGCGGTAGGCGTG-3 and scFv-TM-reverse: 5-CTGCGTGTCCTGGCCCACAGC-3, while the full-length antibody genes were similarly amplified using primers full-length-forward: 5-TGTGATGACCCAAACTCCGC-3 and full-length-reverse: 5-TGCTCTTGTCCACGGTTAGC-3. The products of PCRs were inserted into the T-Vector (Takara) by TA cloning for sequencing.

### Purification of antibodies

The anti-GPCR-full-length variants were produced by co-expressing of heavy chains and light chains using the Expi293F transfection systems (Life Technologies). The cells were harvested 4 days after transfection. The supernatant was collected and purified with a Pierce Protein A Chromatography Cartridge (Thermo Fisher Scientific, Waltham, MA, USA). The anti-GPCR-scFv-Fc antibodies were produced by transfecting Expi293F with the plasmid and following the above-described procedure. The anti-GPCR-scFv-His antibodies were similarly produced using the aforementioned procedure except for utilizing Ni-NTA columns (Amersham Biosciences) for purification.

GFP and RFP were produced by following the procedure of Chen et al. (2016).

### Flow cytometry

Flow cytometers used for cell analysis and cell sorting in this study are FACSCalibur (BD), Influx (BD), FACSAria III (BD), and BriCyte E6 (Mindray, China). The method to sort cells that express a gene of interest was described in transfection and stable cell line establishment.

BriCyte E6 was used to analyze the size of the vesicles, and 100 and 200 nm silica beads (Kisker Biotech GmbH & Co KG, Germany) were used as size references. To analyze the display level and vesicle-binding ability, anti-GPCR-scFv displaying cells were incubated with ETaR-expression vesicles (1:10) and APC-conjugated anti-HA antibody (Pierce, 26183, to demonstrate antibody display level on cells) for 30 min at 4 °C, cells were then washed once with opti-MEM and suspended in cold opti-MEM, and subjected to flow cytometric analysis. Anti-ETaR full-length displaying cells were processed and analyzed similarly except that the antibody display level was revealed with APC-conjugated anti-IgG antibody (BD Pharmingen, 550931, 1:20 in cold opti-MEM medium) instead of anti-HA antibody.

Since the affinity KD values of the antibodies to the individual GPCR ETaR on vesicles cannot be measured with SPR (surface plasmon resonance), the Ki values (different from KD, but also reflects affinity of an antibody against ETaR) were calculated from Ki = IC_50_/(1 + L/K_d_) (Brandt et al. 1987; Cer et al. 2009; Cheng and Prusoff 1973; Nikolovska-Coleska et al. 2004). K_d_ was calculated using GraphPad PRISM 5.0 program and a series of antibody concentrations and corresponding fluorescence signals (geometric mean) on ETaR-expressing CHO cells (dose response curve). IC_50_ was calculated using GraphPad PRISM 5.0 program and a series of competing mutant concentration (not conjugated with fluorescent probe) and corresponding fluorescence signals (geometric mean) on ETaR-expressing CHO cells (inhibition curve), where the fluorescent wild-type antibody at the concentration L was added to the reaction. L was determined according to a dose response curve, usually somewhere around K_d_.

To analyze anti-GPCR-scFv, ETaR-expressing and the negative control cell samples were incubated with purified antibodies at concentrations from 1 to 316 nM with triple dilution increment for 1 h at 4 °C, then washed once and labeled with FITC-conjugated anti-His antibody (Abcam, ab1206, 1:500 in cold opti-MEM medium) for 30 min at 4 °C. After washing, the cells were suspended in opti-MEM, and the signals from antibodies bound to ETaR-expressing cells were quantified using FACSAria III. K_d_ for each clone was determined by fitting the dose-response curve using GraphPad PRISM 5.0 program.

To acquire IC_50_ values of different mutant scFv antibodies, ETaR-expressing and the negative control cell samples were incubated with mutant antibody (not labeled with fluorescent probe) at concentrations ranging between 0.01 and 316 nM with 3-fold dilution increment and FITC-labeled wild-type anti-GPCR at L concentration (refer to “Results” for selected L values) for 1 h at 4 °C. After washing with opti-MEM, the cells were analyzed with FACSAria III for FITC-labeled wild-type anti-GPCR fluorescent signals (geometric mean) bound to the cells. IC_50_ values for different mutant antibodies were derived by fitting the inhibition curve using GraphPad PRISM 5.0 program.

The K_d_ and IC_50_ values for the full-length mutant antibodies were obtained similarly as for mutant single-chain antibodies. The only difference was that PE-conjugated anti-IgG antibody (eBioscience, 12-4998-82) was used to label the full-length mutant antibodies for generating K_d_ values.

## Results

### Interaction of cells displaying antibody and cell-expressing GPCR

**Fig. 1:**
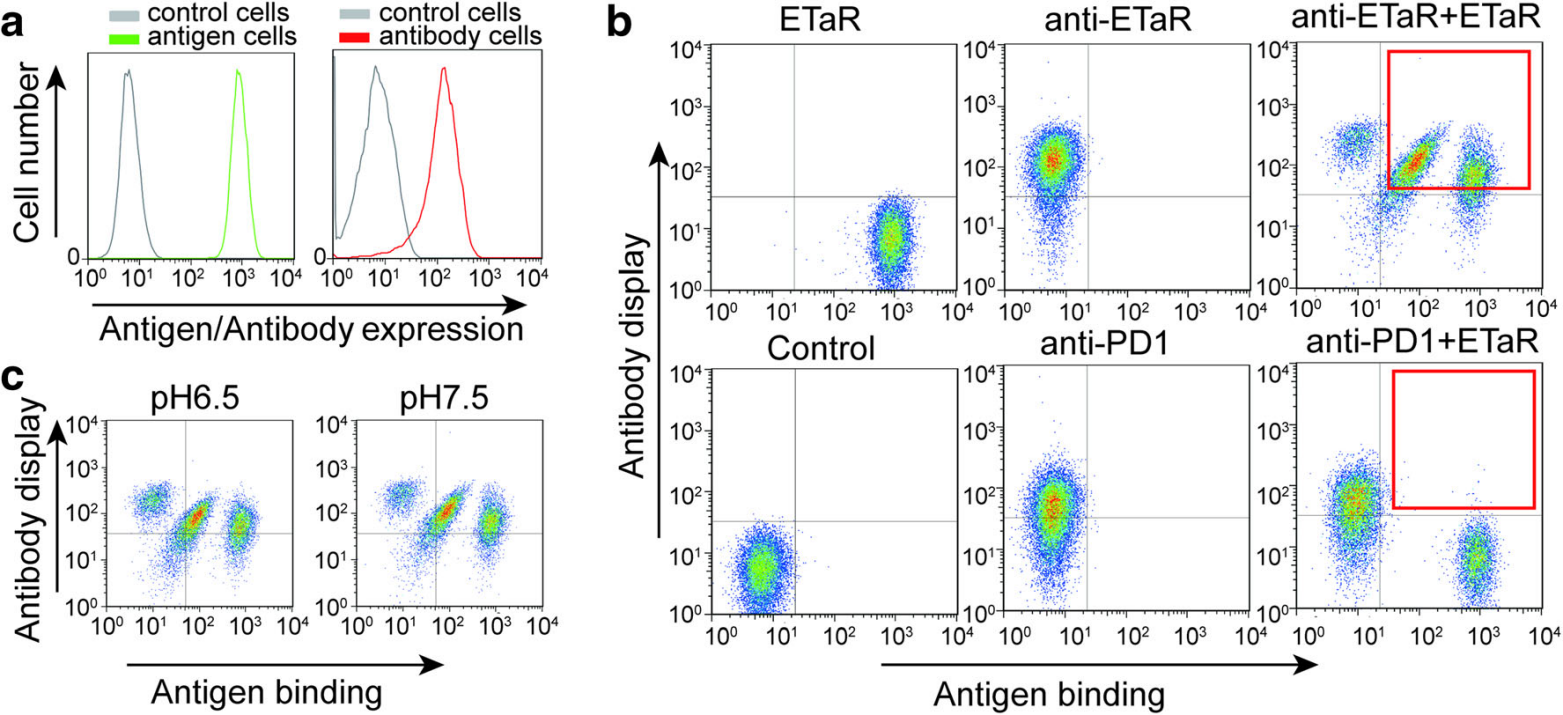
Interaction of the cells displaying anti-ETaR antibody and the cells expressing ETaR. **a** ETaR-GFP expression in CHO cells stably expressed ETaR-GFP (ETaR cells) were detected using flow cytometry (antigen expression, left). CHO cells transfected with anti-ETaR antibody (anti-ETaR cells) were labeled with anti-human-IgG-APC (1:20) and detected using flow cytometry (antibody display, right). **b** ETaR cells were incubated with anti-ETaR cells and CHO cells expressing PD1 (anti-PD1 cells) in a 1:1 ratio. The mixed cells were labeled with anti-human-IgG-APC. Only anti-ETaR cells specifically bound to ETaR cells while the PD1 cells showed no binding. **c** ETaR cells were incubated with anti-ETaR cells in solutions with pH 6.5 and pH 7.5. Antigen binding and antibody display were detected as mentioned above

We intended to establish an antibody affinity maturation platform using CHO cells for displaying antibody and vesicles prepared from CHO cells expressing the GPCR ETaR as probes. Cells might repel each other because of the negative charge on their surfaces. In a previous study (patent CN 101948534 B), we found that a protein displayed on human cells was unable to efficiently interact with its antibody displayed on the inner membrane surface of *E. coli* cells with their outer membrane removed until adjusting the pH of incubation solution significantly lower than the physiological pH 7.5. Thus, the interaction between the cells displaying anti-GPCR and the vesicles displaying GPCR might not be straightforward. To test the feasibility of this platform, we first examined if the cells displaying the antibody and the cells expressing ETaR can efficiently interact. GFP was fused to the C-terminus of ETaR (in cytosol) to trace the cells expressing ETaR. The CHO cells stably expressing ETaR-GFP were prepared by enriching the cells highly expressing ETaR-GFP by flow sorting after transfection with ETaR-GFP and cultured over 10 days (Fig. 1a, left). We transiently transfected CHO cells with anti-ETaR antibody; the majority of the transfected cells positively displayed the antibody (Fig. 1a, right) and the cells were used for the interaction with ETaR-GFP-expressing cells.

The cells expressing ETaR and the cells displaying anti-ETaR antibody were mixed at a 1:1 ratio and incubated for 30 min at 4 °C, then labeled the mixed cells with the APC-conjugated antibody (to emit red fluorescence) against anti-ETaR antibody and subjected to flow cytometric analysis. A portion of the ETaR-positive cells interacted with a portion of the antibody-positive cells (Fig. 1b: rectangle box of the sample file with both anti-ETaR antibody and ETaR-expressing cells). The cells displaying anti-PD1 were used as a negative control and found that only minimal anti-PD1-positive cells interacted with ETaR-positive cells (Fig. 1b: rectangle box of the sample file for anti-PD1 positive cells). These results suggest that many ETaR-positive cells interact with anti-ETaR-positive cells, and the interaction depends on the interaction between ETaR and its antibody. We also tested if the pH of the medium has an influence on the interaction between the cells displaying the antibody and the GPCR and found almost no impact of pH on the interaction (Fig. 1c). Thus, we might be able to prepare small vesicles from the ETaR-expressed cells to perform the maturation of its antibody.

### Vesicles prepared to efficiently monitor the binding ability of anti-ETaR antibody displayed on CHO cells

**Fig. 2:**
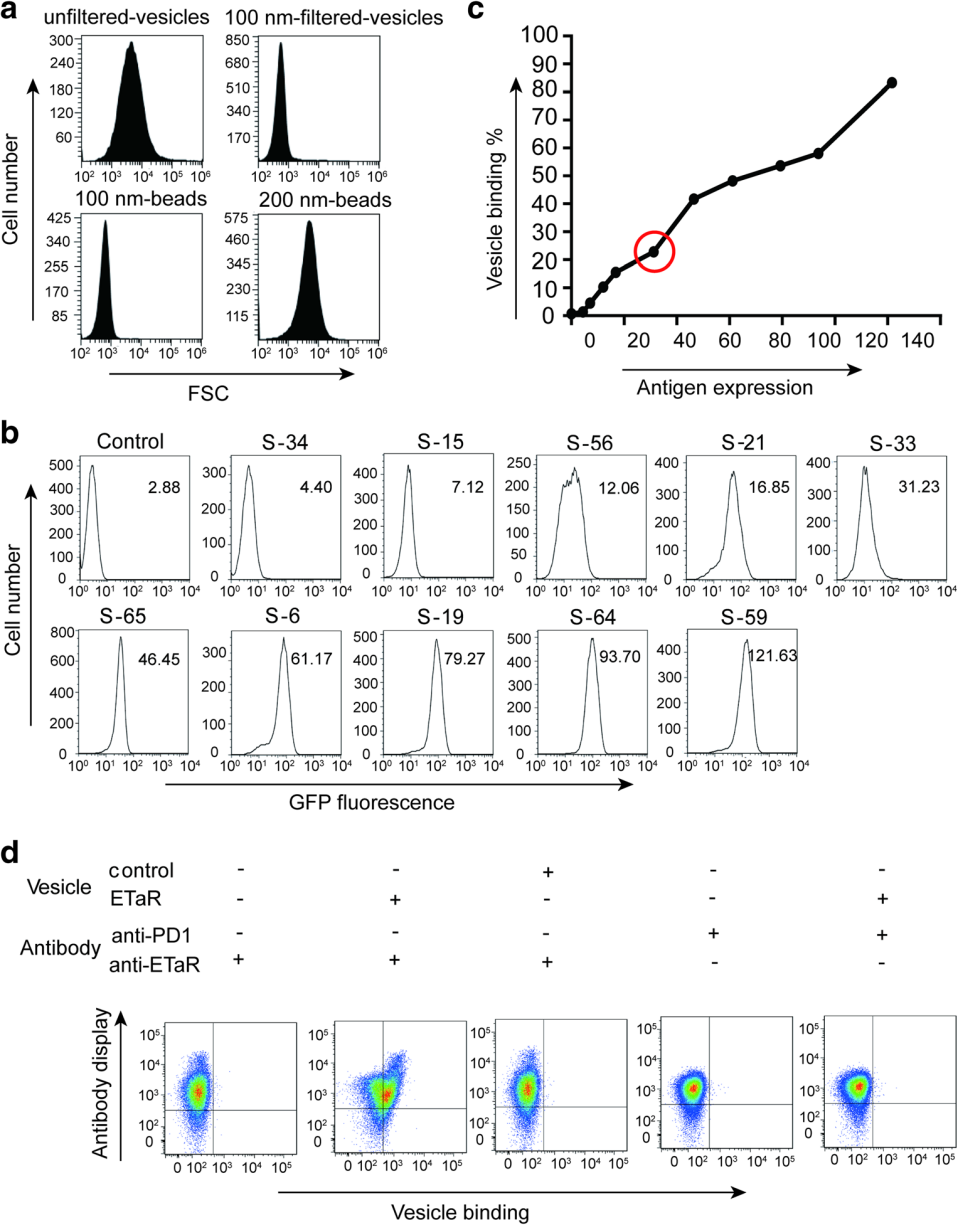
Characterization of the vesicles. **a** Comparison of the size of the vesicles (upper) with the standard beads (lower) according to the FSC (Forward Scattering) parameter detected by flow cytometry (BriCyte E6). The size of 100-nm-filtered vesicles was similar to the 100-nm beads, and the size of unfiltered vesicles was about 200 nm. **b** Single-cell clones with various GFP fluorescence intensity from CHO cells stably expressing ETaR-GFP (ETaR cells) were sorted into 96-well plates using FACSAria III. After 10 days of expansion, the cell clones were detected by a FACSCalibur flow cytometer for the GFP fluorescence intensity. We chose these 10 clones expressing ETaR from the lowest to highest level for further research, and the geometric mean of GFP fluorescence intensity of each clone is shown at the upper right corner of each panel. **c** We prepared unfiltered vesicles expressing various levels of ETaR-GFP from all 11 clones shown in **b**. One tenth of the vesicles prepared by 3 × 10^6^ ETaR-expressing cells were incubated with 1 × 10^6^ CHO cells displaying anti-ETaR antibodies (anti-ETaR cells) for 30 min, and the binding activities were detected using FACSCalibur. Each point represents one cell clone. The geometric mean of GFP fluorescence intensity of each clone is shown on the X-axis, which is also shown in **b**. The binding activities of the vesicles derived from each cell clone are shown on the Y-axis, which was determined by the percentage of the anti-ETaR cells binding with the vesicles. We chose the cell clone S-33 (the dot in the red circle) from these series of clones for preparing vesicles in our following experiments. **d** Characterization of the specificity of the vesicles was derived from the cell clone S-33. We prepared unfiltered vesicles expressing ETaR-GFP (ETaR, derived from clone S-33) and the vesicles only expressing GFP without ETaR (control) as a negative control. Moreover, we used two kinds of cells displaying antibodies: CHO cells displaying anti-ETaR antibodies (anti-ETaR) and CHO cells displaying anti-PD1 antibodies (anti-PD1) as an irrelative control. The vesicles and the cells displaying the corresponding antibodies were incubated for 30 min and detected by FACSAria III. The combination of the vesicles and cells are shown on the top of each panel

Vesicles were prepared by homogenating CHO cells displaying the ETaR molecules and filtering the homogenated cells through a membrane with holes of a designated size (refer to “Materials and methods”). The vesicle size was defined by the pore diameter of filter membrane (Fig. 2a). We hypothesize that multiple GPCR molecules displayed on the membrane of each vesicle would lead to very high avidity and lower the ability to distinguish anti-GPCR antibodies with different affinities, thus posing a challenge to efficiently mature the antibody. To alleviate this difficulty, we isolated CHO cells that expressed the ETaR molecules at low levels. Ten cell clones expressing ETaR from the lowest to highest level were demonstrated here (Fig. 2b). After the vesicles prepared from these cell clones were tested for binding to the antibody-expressed CHO cells, we chose clone S-33 to mature the anti-ETaR antibody (Fig. 2b, c: cycle). The relationship was not linear but positively correlated (Fig. 2c). S-33 has relatively low ETaR expression but strong enough signal of GFP to be detectable by flow cytometry for the sorting of high-affinity antibodies (Fig. S1). Our results confirmed that the vesicles prepared from the clone S-33 specifically bound to the cells displaying anti-ETaR antibody, but not to the cells displaying the irrelevant anti-PD1 antibody (Fig. 2d).

**Fig. 3:**
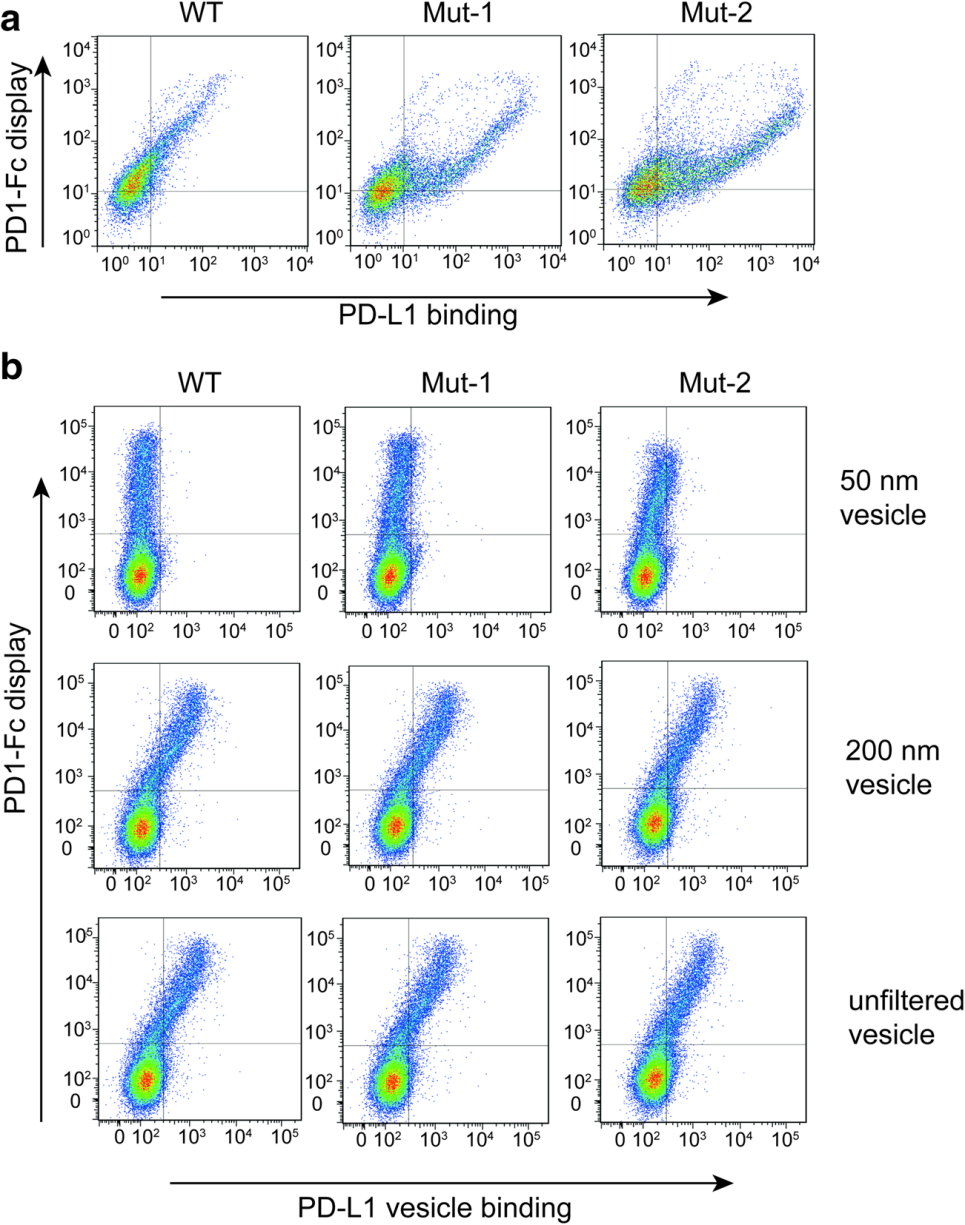
Effects of the size of the vesicles to distinguish proteins with different affinity. **a** CHO cells, which displayed various PD1-Fc proteins with different affinity (WT, Mut-1 and Mut-2), were incubated with soluble PD-L1 proteins for 30 min and then labeled with PE-conjugated anti-His (miltenyi, 130–120-787, 1:300). Affinity analysis performed by flow cytometry showed that the soluble PD-L1 proteins easily differentiated these three PD1-Fc proteins with an affinity of 10-fold apart. **b** CHO cells expressing PD1-Fc proteins as shown in **a** were incubated with 50 nm vesicles, 200 nm vesicles, and unfiltered vesicles prepared from Expi293F cells transiently transfected with PD-L1-GFP for 30 min. Only 50 nm vesicles could distinguish PD1-Fc proteins with different affinities

Another factor that controls the GPCR multivalency on vesicles is the size of the vesicle; the smaller the vesicles, the lower the number of ETaR molecules. The effects of the vesicle size on distinguishing the affinities of antibodies to their antigen were tested using PD1-Fc (program death protein 1) and its affinity-matured clones (our unpublished data) in combination with its ligand PD-L1. As shown in Fig. 3, the free PD-L1 molecule easily distinguished the three different PD1-Fc proteins displayed on CHO cells with about 10-fold affinity apart (Fig. 3a; SPR tested results, our unpublished data). However, the vesicles unfiltered and filtered through 200 nm membrane could hardly differentiate these PD1-Fc proteins displayed on CHO cells. When the size of the vesicle was reduced to 50 nm, the vesicle was able to discriminate the wild-type PD1-Fc molecules from the other two, and also Mut-1 from Mut-2 (Fig. 3b). These results confirm our supposition that small-size vesicles are suitable for antibody maturation and large vesicles are not.

### Maturation of anti-GPCR antibody

**Fig. 4:**
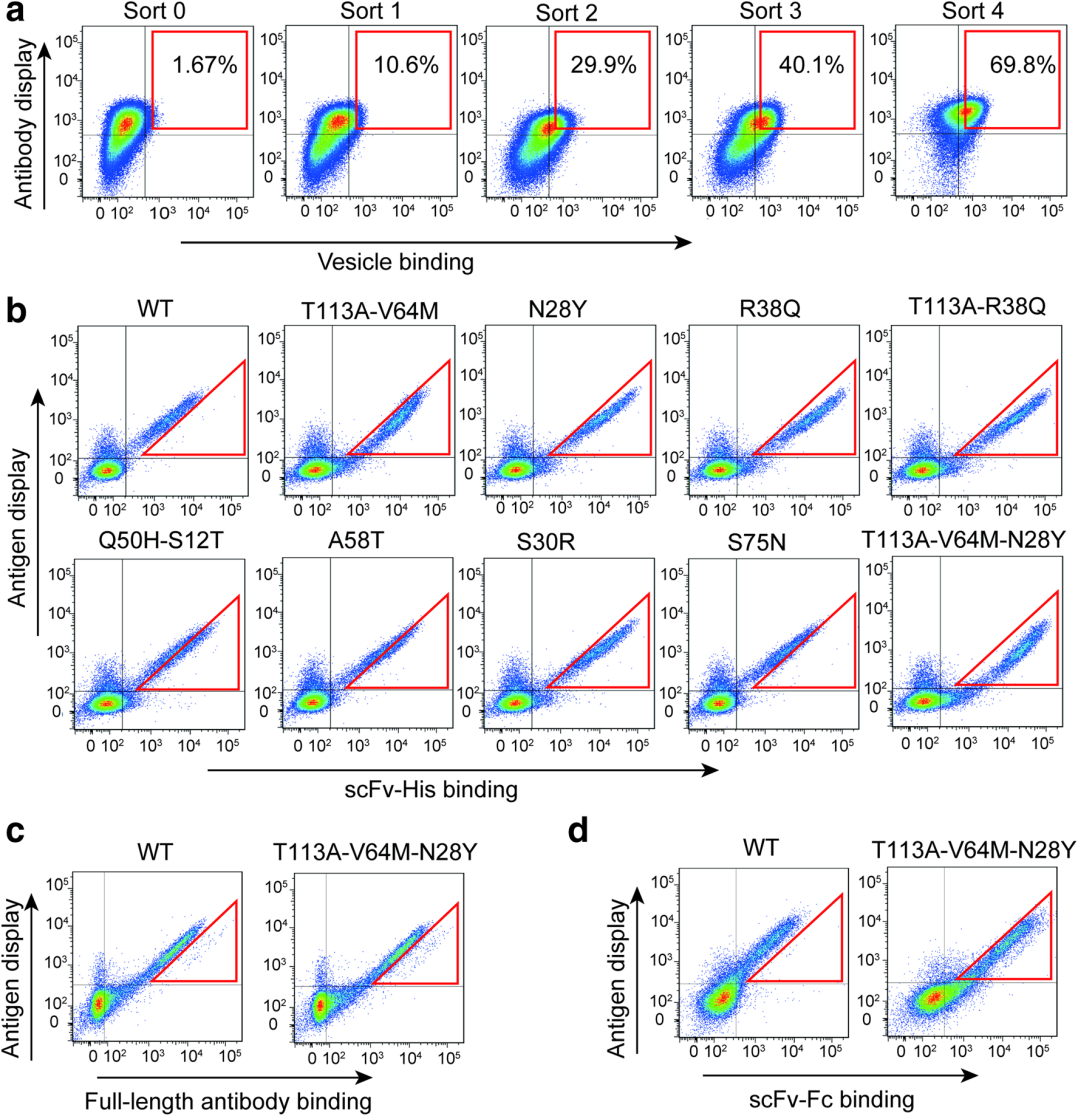
Affinity maturation of single-chain antibodies against ETaR. **a** The single-chain antibody display levels and their vesicle-binding abilities of the sorted cells of each round of maturation with 50 nm ETaR-expressing vesicles. The gates used for quantitative population analysis are shown. **b** Analysis of antigen-binding abilities of anti-ETaR antibodies in their scFv-His format by flow cytometry. The cells expressing ETaR-GFP (antigen) were incubated with purified single-chain antibodies with indicated mutation (labeled on the top of each panel) for 30 min and then labeled with anti-his-PE antibody. The mutant T113A-V64M-N28Y demonstrated the highest affinity compared with the wild-type (WT) antibody. **c** Analysis of antigen-binding abilities of mutant T113A-V64M-N28Y in full-length format. After purifying the full-length antibody of WT and the mutant (T113A-V64M-N28Y), we incubated these two purified antibodies with the cells expressing ETaR-GFP mentioned in **b** for 30 min and then labeled with PE-conjugated anti-IgG antibody (eBioscience, 12-4998-82, 1:400). **d** Analysis of antigen-binding ability of mutant T113A-V64M-N28Y in scFv-Fc format. The flow cytometric measurements were performed similar to **c** after we purified the scFv-Fc fusion proteins of WT and the mutant (T113A-V64M-N28Y)

Antibody affinity maturation using CHO cell display can be performed for either scFv or full-length antibody. We first carried out the maturation of scFv against ETaR. The antibody with an HA tag on the C-terminus was inserted into a specific chromosomal site by gene recombination (refer to “Materials and methodsS16”). The antibody gene can be highly and stably expressed for long-term culture without antibiotic pressure so it is beneficial for highly efficient affinity maturation (Chen et al. 2016). Before the insertion, the antibody gene was codon-optimized to maximize the transcription level (refer to Materials and methods). The transcription level of the targeted gene is directly proportional to the AID-induced mutation rate (Bachl et al. 2001; Fukita et al. 1998; Maul and Gearhart 2010). The higher the mutation rate, the more variant antibody mutants, and the easier it is to obtain clones with high affinity. When the number of cells with the inserted antibody gene reached 300,000 in one well of a 6-well plate, an engineered mouse AID was transfected into them, and the cells proliferated in medium containing neomycin to maintain the AID expression plasmid in cells. When cells grew to about 100 million, the cells were collected and labeled with ETaR-expressed vesicles (50 nm in size) and APC-conjugated anti-HA antibody. Cells displaying antibodies with the highest ETaR-vesicle-binding abilities were enriched by flow cytometry sorting (Fig. 4a: square box for sorted cells of the antibody display level and vesicle-binding ability). Typically, 3000 cells were collected out of 100 million cells. After the collected cells grew to about 300,000, another round of maturation was performed as described above. In total, four rounds of affinity maturation were carried out.

The antibody genes were cloned from the cells after the 4th round of maturation. Thirty out of the 36 sequenced clones contained 1 or 2 point mutations, and there were 16 different mutants (Table S2). These single-chain variable fragment mutant antibodies with His-tag at the C-terminus were synthesized using Expi293F cells (refer to “Materials and methods”), then tested for their ability for binding to ETaR-expressed CHO cells. Since we were unable to prepare individual membrane-free ETaR molecules that process native conformations, we could not compare KD values of the wild-type and generated mutant antibodies using SPR and had to detect the binding of these antibodies to ETaR-expressed cells to calculate K_i_ values for their affinity comparison (refer to “Materials and methods” for detailed description). The mutants N28Y and T113A-V64M demonstrated higher affinities than the wild-type and the other mutants (Fig. 4b and our unpublished data). We combined these two mutants into the T113A-V64 M-N28Y clone, and this antibody showed higher ETaR-binding ability than either one of the original mutants (Fig. 4b). We measured the affinities (K_i_ values) of the wild type and mutants by titrating soluble antibodies and quantifying the ETaR cell binding using flow cytometry (the L in the K_i_ equation was 150 nM). The K_i_ values of the wild type, N28Y, T113A-V64M, and T113A-V64M-N28Y antibodies were 300.4 nM, 75.1 nM, 83.4 nM, and 22.12 nM, respectively (Table 1), confirming the results detected with flow cytometry. Of note, R38Q in scFv form also had significantly higher binding abilities than the wild type (Fig. 4b). However, the combination of R38Q with T113A, with T113A-V64M, and with T113A-V64M-N28Y led to lower (T113A-R38Q) or undetectable antibody expression (T113A-V64M-R38Q and T113A-V64M-R38Q-N28Y), and we only obtained enough protein of T113A-R38Q for affinity analysis among these three mutants. The binding ability of T113A-R38Q scFv-His was not better than R38Q (Fig. 4b), indicating that R38Q is incompatible with T113A-V64M and T113A-V64 M-N28Y in contribution to affinity improvement.

**Table 1:** Kinetic parameters for binding of the ETaR-expression cells to purified scFv-His by flow cytometry

Antibody	K_d_ (nM)	IC_50_ (nM)	K_i_ (nM)
WT	168.7	568.1	300.4
N28Y	83.46	210.1	75.10
T113A-V64M	87.01	227.2	83.40
T113A-V64M-N28Y	22.40	170.4	22.12

We also synthesized these antibodies (wild type and mutant bearing T113A-V64M-N28Y) in the forms of scFv-Fc and full length to examine if the increased affinities derived from the above-described mutant scFv-His are also manifested in double-valent full length and scFv-Fc. As expected, the K_i_ for the full-length wild-type antibody (31.3 nM) was about 10 times smaller than the wild-type scFv-His (300.4 nM). However, the K_i_ for the T113A-V64M-N28Y mutant full-length antibody (15.6 nM) was rather close to that of its mutant scFv-His (22.1 nM). This result was also reflected in flow cytometric files (Fig. 4c). The K_i_ values for the wild-type and mutant scFv-Fc (250.6 and 40.2 nM, respectively) were not much different from those of the scFv-His (300.4 and 22.1 nM, respectively), and the similar result was also shown in Fig. 4d. These results suggest that the conformations of independent light and heavy variable domains in full-length form and linked light and heavy variable domains in scFv-His and scFv-Fc forms are not exactly the same and may confer different affinities. Thus, the mutants obtained from maturating scFv do not necessarily result in the full-length antibodies with expected higher affinities.

**Fig. 5:**
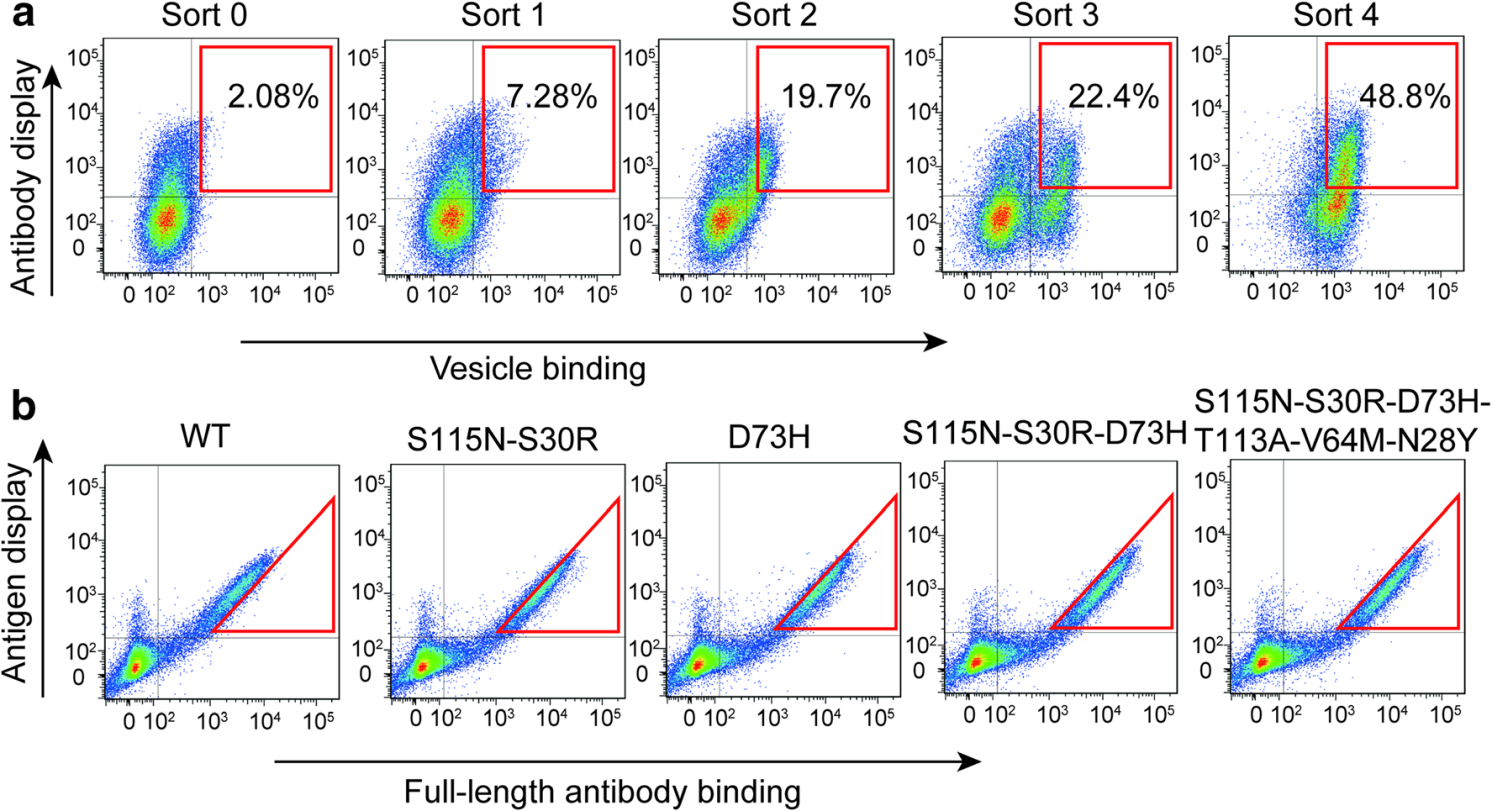
Affinity maturation of full-length antibody against ETaR. **a** The full-length antibody display levels and their antigen-binding abilities of the sorted cells of each round of maturation with 50 nm ETaR-expressing vesicles. The gates used for quantitative population analysis are shown. **b** Analysis of antigen-binding abilities of the screened mutants in their full-length format. The assays were made on cells stably expressing ETaR-GFP. The antigen-expressing cells were incubated with purified mutant antibodies for 30 min and then labeled with anti-human-IgG-PE antibody. The mutant S115N-S30R-D73H demonstrated the highest affinity compared with the wild-type (WT) antibody. We combined these three mutations (S115N-S30R-D73H) with the three mutations (T113A-V64M-N28Y) screened from the maturation of scFv. However, the mutant with these six mutations demonstrated no improvement in affinity compared with the mutant with three (S115N-S30R-D73H) mutations

We wondered if antibody maturation in full length would generate full-length antibodies with higher affinities. We matured the affinity of a full-length antibody following the same procedure described above (Fig. 5a: square box for sorted cells of the antibody display level and vesicle-binding ability). The enriched cells of the third round of maturation contained two separated subpopulations. One subpopulation has a higher display and ETaR-binding ability, and the cells after the fourth round of maturation consisted of only this subpopulation. The full-length antibody genes were cloned from the cells after the fourth round of maturation, 42 out of the 45 sequenced clones contained 1 or 2 point mutations, and there were 18 different mutants (Table S3).

We measured the affinities of the wild type and mutants of the full-length antibody in the same procedure as described above (the L in the K_i_ equation was 13 nM). The affinities of the wild type, D73H, S115N-S30R, and S115N-S30R-D73H antibodies were 31.3 nM, 15.65 nM, 10.17 nM, and 6.2 nM respectively (Table 2). We were surprised to find that when combined, the mutants S115N-S30R-D73H and T113A-V64M-N28Y in their full-length form, the affinity did not improve at all (Fig. 5b).

**Table 2:** Kinetic parameters for binding of the ETaR-expression cells to purified full-length antibodies by flow cytometry

Antibody	K_d_ (nM)	IC_50_ (nM)	K_i_ (nM)
WT	21.52	50.07	31.30
D73H	13.62	30.59	15.65
S115N-S30R	10.85	22.38	10.17
S115N-S30R-D73H	8.266	16.03	6.20

**Fig. 6:**
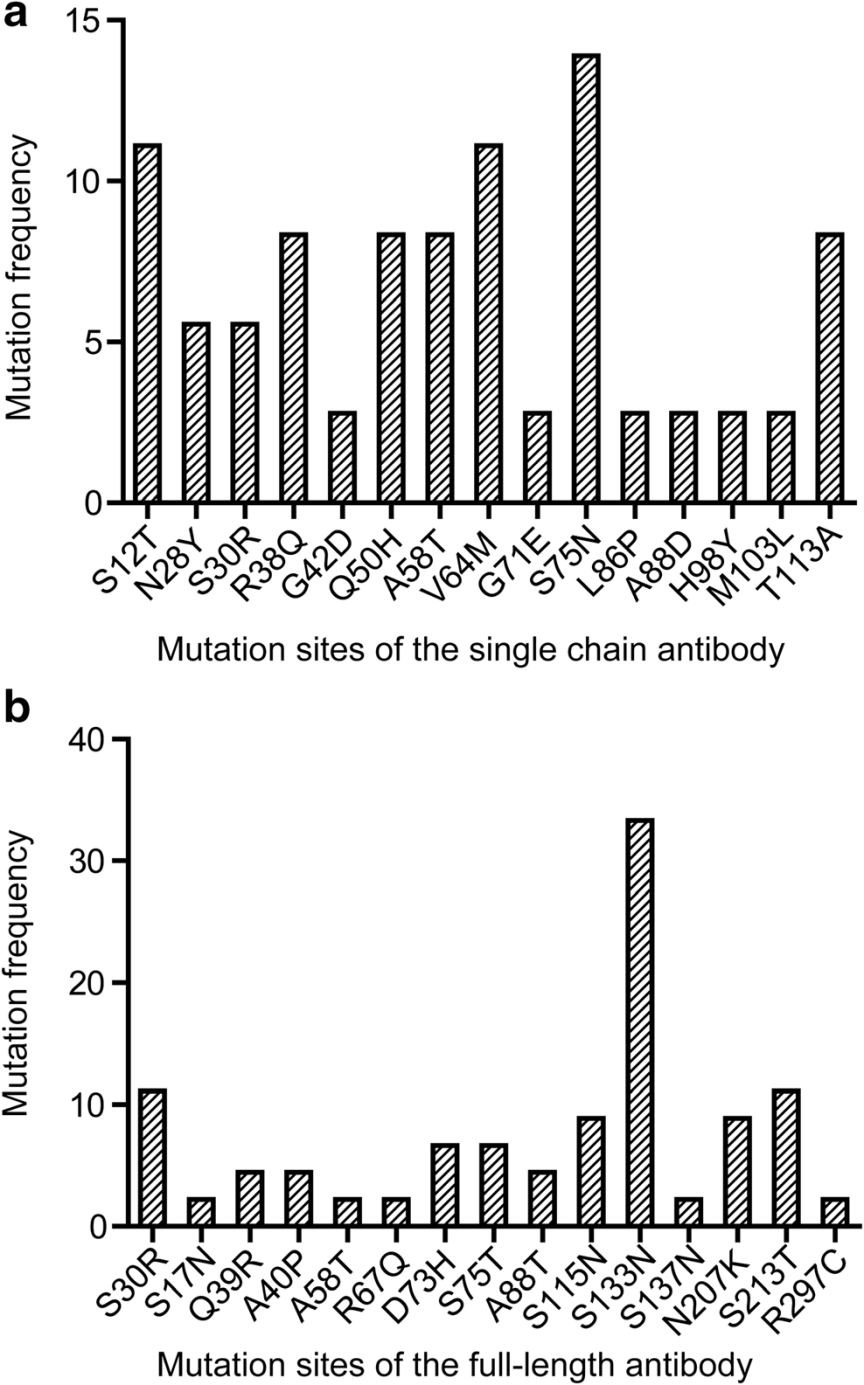
The distribution of mutants derived from affinity maturation in scFv and full-length antibody forms. Hot spot mutants were compiled from the sequences of randomly selected scFv clones (**a**) and full-length antibody clones (**b**), respectively

Results from Tables 1 and 2 as well as Figs. 4, 5, and 6 indicate that antibody mutants with the highest affinities were not the most enriched and that the most point mutations from scFv-His and full-length antibodies were not the same.

## Discussion

In this study, we established a platform to mature the affinity of an antibody against the natively conformed GPCR (ETaR) displayed on the vesicles prepared from cells expressing the GPCR. We carried out the maturation in both scFv and full-length forms and successfully increased the affinity of this antibody (Figs. 4 and 5). This method can be used for affinity maturation of both antibodies against GPCRs and all the other membrane proteins difficult to express without membranes.

GPCR is one of the most important therapeutic drug targets (Hutchings et al. 2017). One of the reasons for scarcity of GPCR antibody drugs is that it is difficult to perform affinity maturation of the selected functional antibodies, which is often required for eventual success in developing therapeutic GPCR-targeting antibodies (Carlin et al. 1999; Maynard et al. 2002; Putnam et al. 2008; Wu et al. 2007). To solve the problem, we combined CHO cell antibody display and vesicle-displaying GPCR to mature the affinity. One underlying reason for our success may be the sufficient difference between the sizes of CHO cells (10,000 nm) and vesicles (50 nm). Yeast display was previously used to mature the affinity of an scFv against the transmembrane protein transferrin receptor (Cho and Shusta 2010), in which detergent-solubilized transferrin receptor was used as a probe to enrich the yeast cells with high binding abilities to the probe. However, detergent solubilization often alters the functional conformation of a GPCR; thus, this method is not always possible to mature an antibody against a specific functional conformation (Geppetti et al. 2015; Hansen et al. 2018; Hotzel et al. 2011). The average sizes of budding yeast and CHO cells are 4000 nm and 10,000 nm respectively, and the difference of their average surface areas is 5.76-fold. Therefore, an affinity maturation by combining yeast display and small vesicle probes could be more challenging, but is worth a trial.

**Fig. 7:**
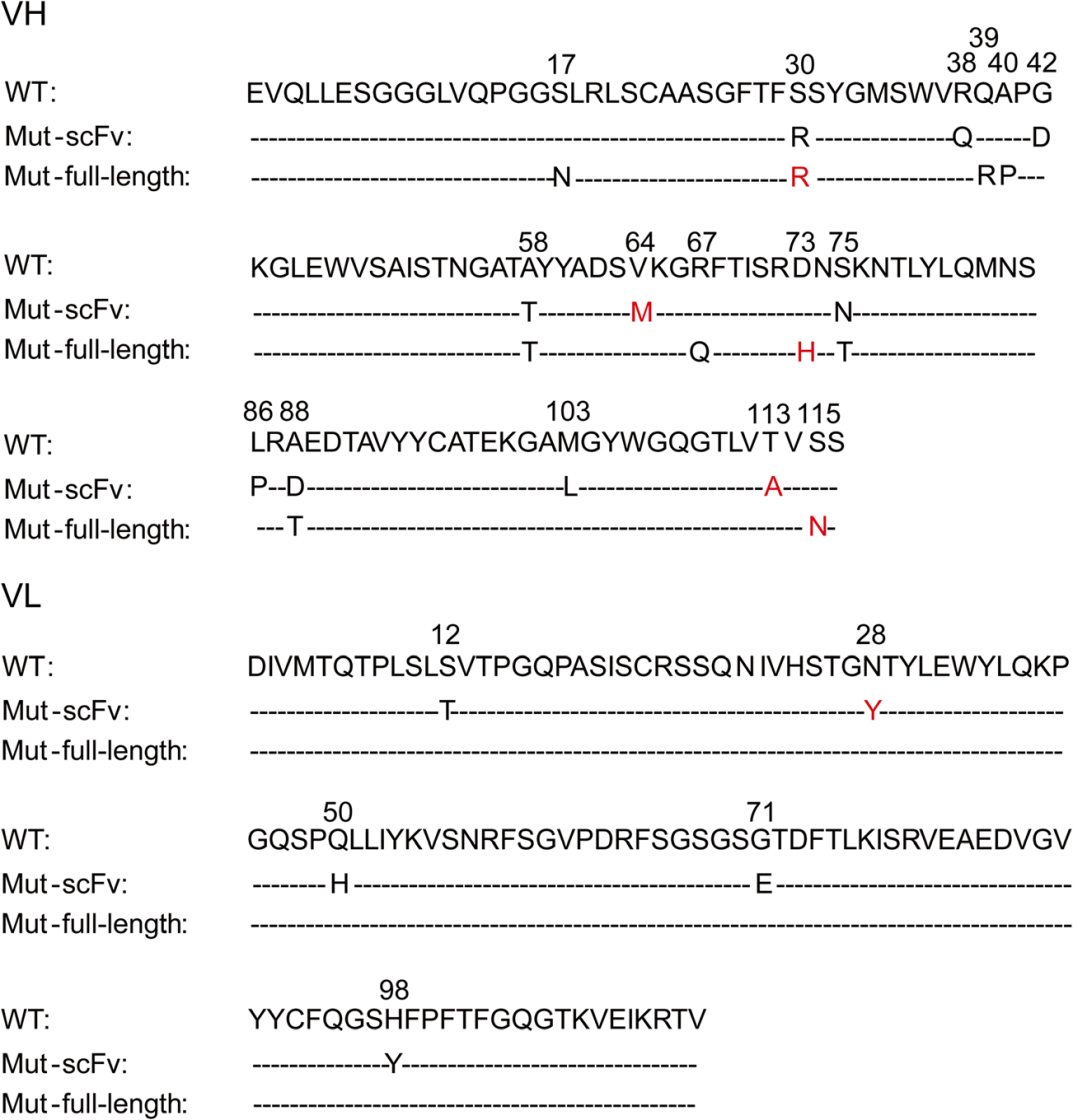
The distribution of amino acid mutants in antibodies derived from four rounds of evolution. The red letters represent the amino acids that contribute to the increase of affinity

It is intuitive to infer that the most enriched antibody clones using our current maturation system are those with the highest affinities. This is true when maturing the affinity of a scFv against an antigen not displayed on vesicles. In one of our previous work to mature antibodies against TNFα, mutants enriched more significantly with each round of maturation (Chen et al. 2016). There were only two mutant clones which were highly enriched after the last round of maturation, and the two clones have higher affinities than any other clones that appeared in the earlier rounds of maturation (Chen et al. 2016). Several other studies also suggested that mutants with the highest affinity are those with the highest enrichment (our unpublished data). However, this is not the case when vesicle-bound GPCR was used, in which many mutant clones existed after the last round of maturation (Tables S2 and S3). The most enriched scFv mutants (S75N, A58T, and Q50H-S12T) had affinities equivalent to or slightly higher than the wild-type antibody, while the clones with the highest affinities were not the most highly enriched (Figs. 6 and 7). Obviously, membrane-bound antigen probes are different from free-antigen probes in affinity maturation. It warrants further investigation on the underlying causes and improvement on the procedure.

When we converted the affinity-improved scFv mutants to full-length antibodies, their affinities (inhibition constants) were not much improved compared to the wild type full-length antibody. The affinity of the best clone (T113A-V64M-N28Y) was only 2-fold of that of the wild-type full-length antibody although the affinity of the same clone in scFv format was greater than 10-fold of the wild-type clone. Similarly in several reported studies, little affinity improvement or even affinity decrease happens when free protein antigens are used for maturing scFv (Muzard et al. 2009; Skrlj et al. 2010). Therefore, in our study, we directly evolved full-length IgG. The best full-length antibody mutant S115N-D73H-S30R had an affinity 5.05-fold of that of the wild type, a better result than that by maturing scFv followed by converting to full-length IgG (2.01-fold of that of the wild type). In addition, we gathered all the point mutations from scFv maturation (T113A, V64M and N28Y) and from full-length antibody maturation (S115N, D73H, and S30R). However, we found that the full-length antibody clone containing the 6 point mutations did not demonstrate stronger antigen-binding ability than the clone S115N-D73H-S30R, suggesting that the mutations from the maturation of scFv and full-length antibody are not additive in this case.

One difficulty we encountered in developing the current maturation procedure was multiple GPCR molecules on vesicles. When antibodies displayed on cells bind to multiple antigens, it results in a significant increase in apparent affinity (avidity) (Deng et al. 1995; Griffiths et al. 1993; Holliger et al. 1993; Kortt et al. 1994; Whitlow et al. 1994). Reduction of the vesicle size and number of GPCR molecules per vesicle can decrease the avidity and increase the ability to monitor the different affinities of different antibody clones displayed on cells.

Due to the multiple GPCR molecules on the vesicles, the GPCR-displaying vesicles showed apparent low sensitivity inevitably in distinguishing different affinity antibodies compared with soluble protein antigens. This was clearly demonstrated when three different sizes (50, 200, and unfiltered) of vesicles were used to monitor the difference of the three PD1-Fc mutants with 10-fold affinity apart (Fig. 3). It can hardly distinguish these three PD1-Fc mutants when 200 nm or unfiltered vesicles were used; thus, it would be impossible to use the large-sized vesicles to perform the affinity maturation. In this study, we used 50 nm size vesicle for affinity maturation of anti-ETaR antibody, which showed higher sensitivity. In order to maintain the correct conformation of antigen molecules in vesicles, the size of the vesicles cannot be below 30 nm (Wan et al. 2017). The size of a full-length antibody is about 10 nm. It is worthwhile to test whether the 30 nm vesicle would work, and we may establish a more efficient antibody evolution system against GPCRs. Although the smaller vesicles confer better sensitivity to distinguish different cells displaying binders with different affinities to ligand (Fig. 3b), considering that the increased curvature of the membrane surface of small vesicles could affect conformation of the membrane protein antigen, the best size of vesicles for each antigen should be found out before performing the formal maturation procedure. One of the criteria that can be used to show that the GPCRs on small vesicles are not functionally compromised is to test if these GPCRs can be phosphorylated in response to the addition of their corresponding ligands (Prihandoko et al. 2015).

Considering the effect of the number of GPCR molecules per vesicle on the maturation efficiency, we chose to use a vesicle displaying a low but sufficient number of GPCR molecules which can just be detected when starting the maturation process (refer to the sort 0 file in Fig. 4). It is possible that even a lower number of GPCR molecules per vesicle can be used and may offer a better sensitivity to monitor a small affinity difference between antibody mutants. However, this will not be feasible to label vesicles by GPCR-GFP fusion protein because the signal of a very few number of GPCR-GFP per vesicle will be too weak for detection. To overcome this problem, we tested whether fluorescence protein and a small molecule dye could be directly added into the vesicles being prepared instead of preparing cell clones expressing GPCR-fluorescence protein. GFP or RFP synthesized in *E. coli* was purified and added to the ETaR-expressed CHO cells in suspension buffer before being homogenated to form vesicles. The unfiltered vesicles were incubated with the negative control CHO cells and the CHO cells displaying anti-ETaR antibody. Clearly, vesicles containing GFP or RFP specifically bound to the antibody-displayed cells, but only few vesicles bound to the negative cells (Fig. S2). We also tested if FITC can be added into vesicles as for fluorescence proteins, and we found that a few cells were much brighter than the majority of cells regardless that there were negative or antibody-displayed cells (our unpublished data), making affinity maturation impossible. It will be worthwhile to test other small fluorescence molecules for this purpose in the future. In conclusion, free fluorescence protein in high concentration can be wrapped inside the vesicles to provide strong enough signals for antibody affinity maturation. It warrants trying this strategy to prepare vesicles to improve the efficiency of the platform.

## Electronic supplementary material

ESM 1 (PDF 395 kb)
