# Supplementary material for: In vitro affinity maturation of antibody against membrane-bound GPCR molecules
Source: Appl Microbiol Biotechnol. 2019 Jul 29;103(18):7703–17. doi: 10.1007/s00253-019-10030-x (PMC6719327; doi:10.1007/s00253-019-10030-x)
Supplement: Supplementary file 1 — (PDF 395 kb) [file 253_2019_10030_MOESM1_ESM.pdf]

Applied Microbiology and Biotechnology

In vitro affinity maturation of antibody against membrane-bound GPCR molecules

Jie Wang[1, 2], Lili An[1], Yun Zhao[1], Cheng Zhang[3], Shengnan Li[3], Chen Ye[1], Shuqian Jing[3, *], and Haiying Hang[1, 2, *]

[1]Key Laboratory for Protein and Peptide Pharmaceuticals, National Laboratory of Biomacromolecules, Institute of Biophysics, Chinese Academy of Sciences, Beijing 100101, China.

[2]University of Chinese Academy of Sciences, Beijing 100039, China.

[3]Gmax Biopham LLC, Hangzhou 310052, China.

*To whom correspondence should be addressed.

Prof. Haiying Hang, Tel/Fax: +86-10-64888473; Email: hh91@ibp.ac.cn. Correspondence may also be addressed to Prof. Shuqian Jing, Tel/Fax: +86-0571-86633907; Email: sjing@gmaxbiopharm.com

Fig. S1

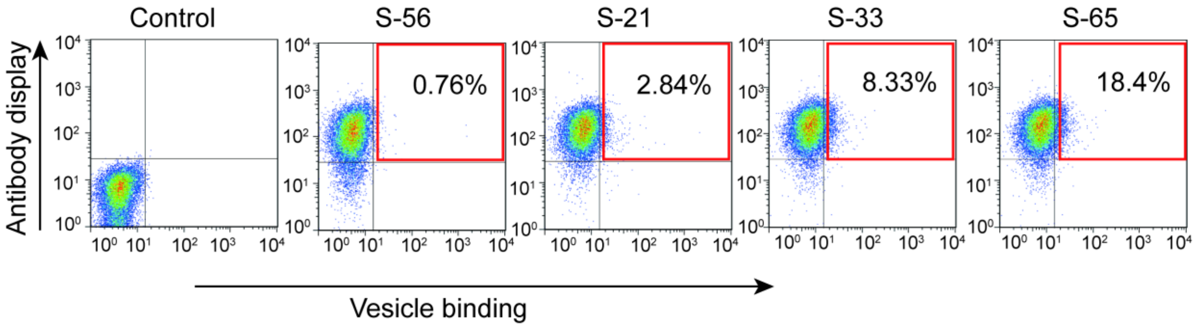

Fig. S1 Characterization of 50 nm vesicles for binding the anti-ETaR single chain antibody. We prepared several 50 nm vesicles expressing various low levels of ETaR-GFP from the clones respectively shown in Fig. 2b. The gates used for the analysis of the positive binding signal are shown. One tenth of the vesicles prepared by 3×10^6 ETaR-expressing cells were incubated with 1×10^6 CHO cells displaying anti-ETaR single chain antibodies for 30 min and the binding activities were detected using FACSAria III

Fig. S2

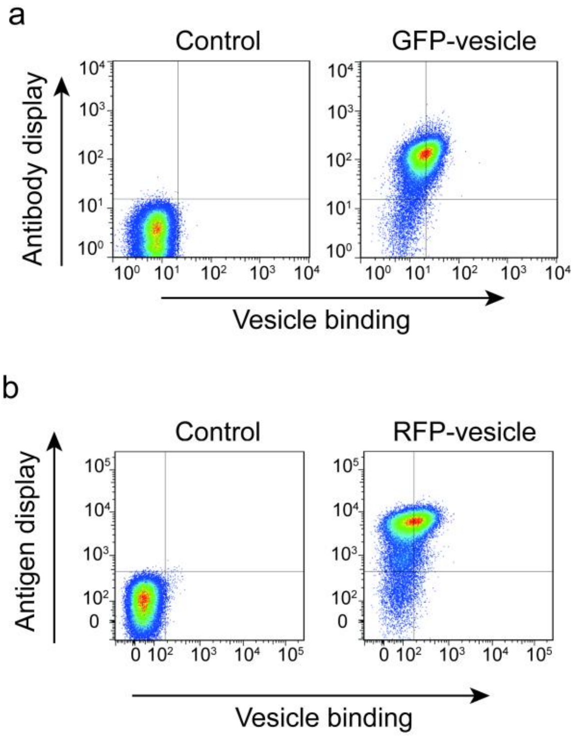

Fig. S2 Alternative strategy of vesicle preparation. We prepared vesicles by directly adding the fluorescence proteins into the vesicles from the cells expressing ETaR protein (without GFP). **a** GFP proteins were purified in *E. coli*. After collecting the ETaR-expressing CHO cells (4×10^7), we added 2

mg GFP proteins to the ETaR-expressing cells in suspension buffer before being homogenated to form vesicles (GFP-vesicle). GFP vesicles were incubated with the negative control CHO cells and the CHO/dhFr$^-$ cells displaying anti-ETaR antibody for 30 min respectively, and detected by flow cytometry. **b** RFP proteins were purified in *E. coli.* After collecting ETaR-expression cells (4×10^7), we added 3 mg of RFP protein to the cells in suspension buffer before homogenating to form vesicles (RFP-vesicle). RFP vesicles were incubated with the negative control CHO/dhFr$^-$ cells and the CHO cells displaying anti-ETaR antibody for 30 min respectively, and detected by flow cytometry

Table S1 Plasmids and primers used in this study

Plasmids	Primers	Sequences
pFRT-anti-GPCR-scFv	scFv-*Eco*R1-P1	5’-ATACGCGAATTCATGACCCGGCTGACCGTGCTGGCCCTGCTGGCCGGCCTGCTGGCCTC-3’
	Overlap-scFv-P2	5’-GGCCCTGCTGGCCGGCCTGCTGGCCTCCTCCAGGGCCGAAGTGCAGCTGCTGGAATC-3’
	Overlap-scFv-P3	5’-CGTTAGCTCTGGTGGTGGTGGTTCTGGTGGTGGTGGTTCTGGCGGCGGCGGCTCCGACATCGTGAT-3’
	Overlap-scFv-P4	5’-ATCACGATGTCGGAGCCGCCGCCGCCAGAACCACCACCACCAGAACCACCACCACCAGAGCTAACG-3’
	scFv-*Xho*I-P5	5’-ATACGCCTCGAGAGCATAATCTGGAACATCATATGGATACACGGTTCTCTTGATTTCCAC-3’
pFRT-anti-GPCR-full-length	LC-*Eco*RI-P1	5’-ATACGCGAATTCGCCACCATGGACTTTGGGCTGAGCTG-3’
	LC-*Eco*RV-P2	5’-CTACGCGATATCCTAACACTCTCCCCTGTTGAAGCTC-3’
	HC-*Nhe*I–P3	5’-ATACGCGCTAGCGCCACCATGGACTTTGGGCTGAGCTG-3’
	HC-*Xho*I–P4	5’-ATACGCCTCGAGACCCAGAGACAGGGAGAGGCTCTTC-3’
pCEP4-anti-GPCR-scFv-His	His-*Hin*dIII-P1	5’-ATACGCAAGCTTGCCACCATGACCCGGCTGACCGTGCTGGCCC-3’
	His-*Xho*I-P2	5’-ATACGCCTCGAGCTATCAGTGATGATGATGATGATGGTGATGATGATGATGATGACTTCCTCCTCCTCCCACGGTTCTCTTGATTTCCACCTTG-3’
pCDNA3.1(+)-anti-GPCR-full-length-LC	LC-*Eco*RI-P1	5’-ATACGCGAATTCGCCACCATGGACTTTGGGCTGAGC-3’
	LC-*Xho*I-P2	5’-ATACGCCTCGAGCTAACACTCTCCCCTGTTGAAGCTC-3’
pCDNA3.1(+)-anti-GPC	HC-*Cla*I-P1	5’-ATACGCATCGATTAGGCCACCATGGACTTTGGGCTGAGC-3’

R-full-length-HC	HC-*Xho*I-P2	5'-ATACGCCTCGAGCTAACCCAGAGACAGGGAGAGG CTC-3'
pCEP4-anti-GPCR-scFv-Fc	*Hin*dIII-P1	5'-ATACTCAAGCTTGCCACCATGACCCGGCTGACCGT GCTGGC-3'
	Overlap-P2	5'-GGCATGGGGGACCATATTTGGACACGGTTCTCTTG ATTTCCACCTTG-3'
	Overlap-P3	5'-GGTGGAAATCAAGAGAACCGTGTCCAAATATGGT CCCCCATGCCCAC-3'
	*Xho*I-P4	5'-AGACGCCTCGAGTTATTAACCCAGAGACAGGGAG AG-3'
pET28a(+)-GFP	GFP-*Sac*I-P1	5'-ATCCGCGAGCTCATGGTGAGCAAGGGCGAGGAG-3 '
	GFP-*Xho*I-P2	5'-ATCCGCCTCGAGTCTTGTACAGCTCGTCCATG-3'
pET28a(+)-RFP	RFP-*Bam*HI-p1	5'-ATACGCGGATCCATGGCCTCCTCCGAGGACG-3'
	RFP-*Hin*dIII-p2	5'-ATACGCAAGCTTTTAGGCGCCGGTGGAGTGG-3'
pCEP4-PD-L1-GFP -TM	PD-L1-*Hin*dIII-P1	5'-TGACTAAGCTTGCCACCATGACCCGGCTGACCGTG CT-3'
	PD-L1-*Xho*I-P2	5'-CTGTCCTCGAGCTTGTACAGCTCGTCCATGC-3'
pCEP4-PD1-Fc	SP-*Hin*dIII-P1	5'-ATACGCAAGCTTGCCACCATGACCCGGCTGACCGT GCTGGCCC-3'
	PD1-Overlap-P 2	5'-GGGCATGTGTGAGTTTTGTCACAAGATTTGGGCTC TTCTG CGCGCCTCTC GGTCACCC-3'
	Fc-Overlap-P3	5'-GGGTGACCGAGAGGCGCGCAGAAGAGCCCAAATC TTGTGACAAAACTCACACATGCCC-3'
	Fc-*Bam*HI-P4	5'-ATACGCGGATCCTTATTATTTA CCCGGAGACA GGGAGAGGC-3'

Table S2 Mutations observed from the 4th round of the single chain antibody affinity maturation

Mutants	Sort4[a]
S75N	4/36[b]
A58T	3/36
Q50H-S12T	3/36
T113A-V64M	3/36
R38Q	2/36
S30R	2/36
N28Y	2/36
T113A-R38Q	1/36
A88D	1/36
M103L	1/36
H98Y	1/36

S75N-V64M	1/36
L86P	1/36
S12T	1/36
G71E	1/36
G42D	1/36

a The 4th round of the single chain antibody affinity maturation after which the mutations on the antibody gene were revealed by sequencing

b The number of mutant clones / The numbers of sequenced clones

Table S3 Mutations observed from the 4th round of the full-length antibody affinity maturation

Mutants	Sort4 [a]
S133N	4/45 [b]
S133N-N207K	4/45
S213T	5/45
S133N-A171V	3/45
S133N-R297C	3/45
S75T	3/45
D73H	3/45
S115N-S30R	4/45
A88T	2/45
A40P	2/45
Q39R	2/45
S115N	1/45
S30R	1/45
S17N	1/45
R67Q	1/45
A58T	1/45
R297C	1/45
S133N-S137N	1/45

a The 4th round of the full-length antibody affinity maturation after which the mutations on the antibody gene were revealed by sequencing

b The number of mutant clones / The numbers of sequenced clones
